# Anti-edema and antioxidant combination therapy for ischemic stroke via glyburide-loaded betulinic acid nanoparticles

**DOI:** 10.7150/thno.35791

**Published:** 2019-09-21

**Authors:** Gang Deng, Chao Ma, Haitian Zhao, Shenqi Zhang, Jun Liu, Fuyao Liu, Zeming Chen, Ann T. Chen, Xin Yang, Jonathan Avery, Pan Zou, Fengyi Du, Keun-poong Lim, Daniel Holden, Songye Li, Richard E. Carson, Yiyun Huang, Qianxue Chen, W. Taylor Kimberly, J. Marc Simard, Kevin N. Sheth, Jiangbing Zhou

**Affiliations:** 1 Department of Neurosurgery, Yale University, New Haven, CT, 06510, USA.; 2 Department of Neurosurgery, Renmin Hospital of Wuhan University, Wuhan 430060, China.; 3 College of Biological Sciences and Biotechnology, Beijing Forestry University, Beijing 100083, China.; 4 School of Chemistry and Chemical Engineering, Harbin Institute of Technology, Harbin 150090, China.; 5 Department of Biomedical Engineering, Yale University, New Haven, CT, 06510, USA; 6 PET Center, Department of Radiology and Biomedical Imaging, Yale University, New Haven, CT, 06510, USA; 7 Department of Neurology, Division of Neurocritical Care, Massachusetts General Hospital, Boston, MA, 02114, USA.; 8 Department of Neurosurgery, University of Maryland School of Medicine, Baltimore, MD, 21201, USA.; 9 Department of Neurology, Yale University, New Haven, CT, 06510, USA.

**Keywords:** ischemic stroke, betulinic acid, glyburide, antioxidant, combination therapy

## Abstract

Stroke is a deadly disease without effective pharmacotherapies, which is due to two major reasons. First, most therapeutics cannot efficiently penetrate the brain. Second, single agent pharmacotherapy may be insufficient and effective treatment of stroke requires targeting multiple complementary targets. Here, we set to develop single component, multifunctional nanoparticles (NPs) for targeted delivery of glyburide to the brain for stroke treatment.

***Methods:*** To characterize the brain penetrability, we radiolabeled glyburide, intravenously administered it to stroke- bearing mice, and determined its accumulation in the brain using positron emission tomography-computed tomography (PET/CT). To identify functional nanomaterials to improve drug delivery to the brain, we developed a chemical extraction approach and tested it for isolation of nanomaterials from *E. ulmoides*, a medicinal herb. To assess the therapeutic benefits, we synthesized glyburide-loaded NPs and evaluated them in stroke- bearing mice.

***Results:*** We found that glyburide has a limited ability to penetrate the ischemic brain. We identified betulinic acid (BA) capable of forming NPs, which, after intravenous administration, efficiently penetrate the brain and significantly reduce ischemia-induced infarction as an antioxidant agent. We demonstrated that BA NPs enhance delivery of glyburide, leading to therapeutic benefits significantly greater than those achieved by either glyburide or BA NPs.

***Conclusion:*** This study suggests a new direction to identify functional nanomaterials and a simple approach to achieving anti-edema and antioxidant combination therapy. The resulting glyburide- loaded BA NPs may be translated into clinical applications to improve clinical management of stroke.

## Introduction

Stroke is a leading cause of mortality and morbidity worldwide 1_._ In particular, ischemic stroke is the most common type of stroke and accounts for about 87% of all cases 1. Despite its high prevalence, there are limited pharmacotherapies targeting brain tissues for stroke. Currently, the only FDA-approved therapeutic for clinical management of stroke is intravenous administration of tissue-type plasminogen activator (tPA), which functions by dissolving clots in blocked blood vessels 2. However, because tPA must be administered to patients within four and a half hours of symptom onset in order to be effective, many patients are ineligible for this treatment and eventually develop fatal complications 2.

Two factors complicate the development of pharmacological therapies for stroke treatment. First, the brain possesses the blood-brain barrier (BBB), which prevents the penetration of most agents to the brain 3. Although the BBB is partially disrupted after ischemic insult, the degree of disruption may not be sufficient to allow delivery of pharmacologically significant quantities of drugs for effective treatment 4, 5. Second, there is a lack of effective therapeutic regimens. Over the past few decades, many pharmacological therapeutics have been developed to target various pathological processes of stroke, such as cerebral edema, oxidative stress, excitotoxicity, and inflammation 6-8. However, due to limited success in clinical trials, none of these therapeutics have received the FDA approval for clinical use thus far 6-9. One of the most promising tested agents was glyburide, an antagonist to the sulfonylurea receptor 1 (SUR1)-transient receptor potential melastatin 4 (SUR1-TRPM4) cation channel that mediates cerebral edema 10. In the recently completed phase II Glyburide Advantage in Malignant Edema and Stroke (GAMES-RP) study, we demonstrated that intravenous infusion of glyburide significantly improved patient survival. However, the GAMES-RP trial did not meet its primary endpoint to prevent decompressive craniectomy or significantly increase the proportion of patients with favorable clinical outcome 11. Thus, it is likely that a single therapeutic agent is insufficient, and effective treatment of stroke requires simultaneously targeting multiple complementary targets 8, 9.

Herbal medicine has been widely used for clinical management of various diseases in human history 12. A recent analysis suggests that over 200 medicinal herbs might be effective for stroke treatment 13, 14. However, it is unknown how the bioactive compounds in these herbs exert their pharmacological activities on diseases in the brain, as most compounds isolated from herbs are known to have a limited ability to penetrate the brain 15. We hypothesized that, in addition to containing bioactive compounds, certain medicinal herbs also have natural nanomaterials that can encapsulate and deliver drugs to the brain through formation of nanoparticles (NPs).

To test the hypothesis, we developed a chemical extraction approach and studied* E. ulmoides,* an herb often used in Traditional Chinese Medicine (TCM) for stroke treatment 14, 16. Through this approach, we identified betulinic acid (BA), a natural compound that forms NPs. We found that BA NPs were capable of efficiently penetrating the brain and function as antioxidant agents to effectively promote functional recovery in stroke-bearing mice induced by middle cerebral artery occlusion (MCAO). We showed that BA NPs can be employed as a drug carrier to significantly enhance the delivery of glyburide, which has a limited ability to penetrate the ischemic brain as determined by positron emission tomography-computed tomography (PET/CT), resulting in therapeutic benefits greater than those achieved by either glyburide or BA NPs alone. Our study suggests a new direction to identify functional nanomaterials as well as a promising approach to achieving anti-edema and antioxidant combination therapy for ischemic stroke via a simple formulation.

## Methods

### Cell culture

HEK293 cells and hCMEC/D3 cells were obtained from American Type Culture Collection (ATCC). Normal human astrocytes (NHA) cells were purchased from Lonza. Cells were maintained in DMEM supplemented with 10% v/v fetal bovine serum and PSG, all from ThermoFisher, in a pre-humidified atmosphere at 37 °C containing 5% v/v CO_2_.

### Synthesis of ^11^C-labeled glyburide

**Figure 1:**
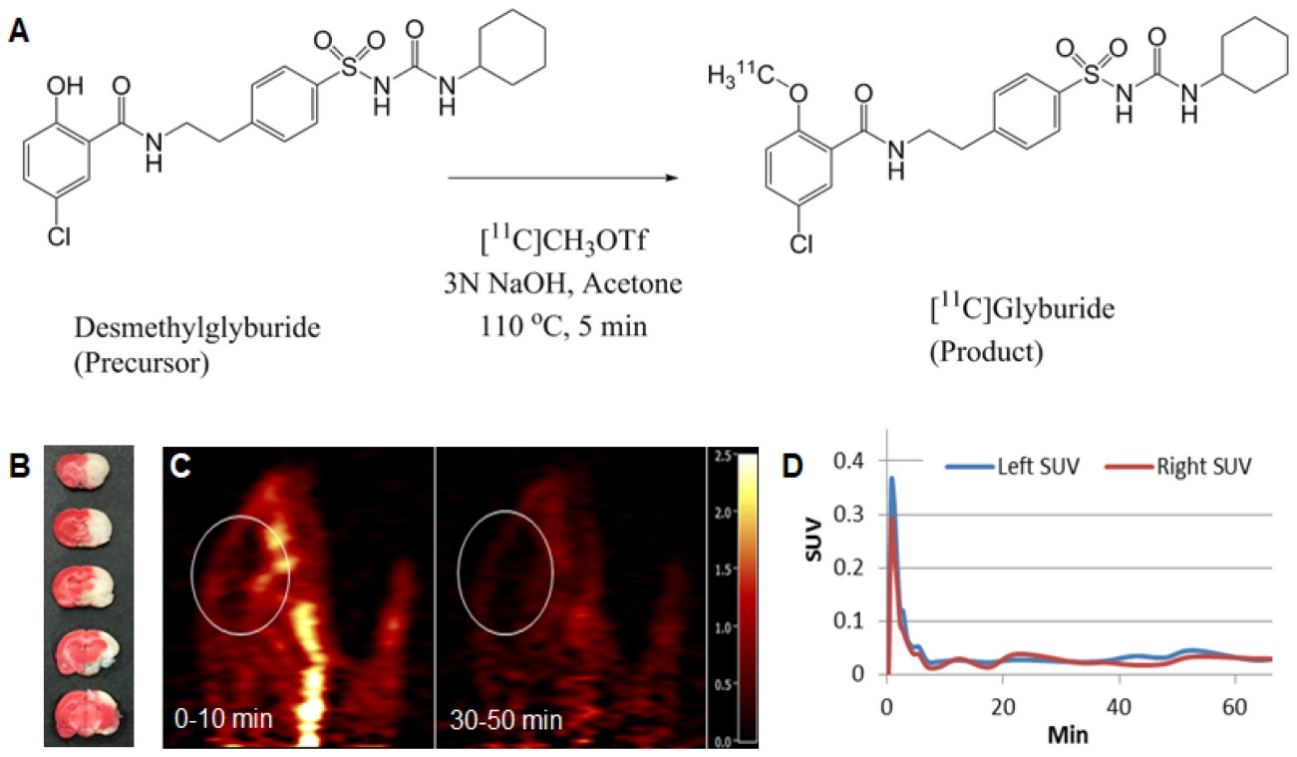
PET imaging of ^11^C-labeled glyburide in MCAO rats. (A) Radiosynthesis of ^11^C-glyburide. (B) TTC staining confirmed the ischemic stroke. (C) Summed images of the brain (marked) at the indicated time frames. (D) SUV activity with time for left (normal) and right (ischemic) hemispheres.

[^11^C]Glyburide was synthesized by [^11^C]-methylation of its desmethyl precursor (Figure 1A) with [^11^C]MeOTf in a TRACERLab^TM^ FxC automated synthesis module (GE Medical Systems). [^11^C]CO_2_ was produced via the ^14^N(p,α)^11^C reaction in a PETtrace cyclotron (GE, Milwaukee, WI) by bombardment of a target filled with 1% oxygen in nitrogen. [^11^C]CO_2_ was the reacted with hydrogen at 400 ^o^C under a nickel catalyst to afford [^11^C]CH_4_, which was converted to [^11^C]CH_3_I by a gas phase reaction with iodine. [^11^C]CH_3_I was then swept through the silver triflate column at 190 ^o^C and the resulting [^11^C]CH_3_OTf was bubbled into the solution of desmethyl glyburide (1.0 mg) in acetone (0.4 mL) and 3 N NaOH (8 µL) cooled at -10 ºC until activity peaked. The reaction mixture was heated at 110 °C for 5 min, cooled to room temperature, diluted with 1.0 mL of 0.1 % trifluoroacetic acid (TFA) and injected onto the semi-preparative HPLC column (Luna C18(2), 10 µm, 10 × 250 mm). The column was eluted with a mobile phase of 55 % MeCN and 45 % 0.1 M TFA solution at a flow rate of 5 mL/min. The radioactivity fraction eluting between 10-11 min was collected, diluted with a solution of 300 mg of United States Pharmacopeia (USP) grade ascorbic acid in 40 mL of deionized (DI) water, and then loaded onto a Waters Classic C18 SepPak cartridge. The SepPak was rinsed with a solution of 10 mg USP ascorbic acid in 10 mL of DI water, and dried with air. The product was eluted off the SepPak with 1 mL of USP absolute ethanol (Pharmco-AAPER) followed by a solution of 3 mg USP ascorbic acid in 3 mL of USP saline (American Regent). The resulting solution was passed through a sterile 0.22 µm membrane filter (33 mm, Millex^®^ GV, Millipore) into a sterile vial pre-charged with 7 mg of USP ascorbic acid in 7 mL of USP saline.

Radiochemical purity and molar activity of [^11^C]glyburide was determined by HPLC analysis using an Shimadzu Prominence system equipped with a LC-20AT pump, a Luna C18 column (5 µm, 4.6 mm x 250 mm), and a SPD-20A UV/Vis detector connected in series with a Bioscan Flow-Count gamma-detector. The system was eluted with a mobile phase of 53% MeCN with 47% of 0.1% TFA at a flow rate of 2 mL/min. The eluent was monitored for radioactivity and UV absorbance at 230 nm (*t*_R_ = 7.5 min for [^11^C]glyburide). The molar activity for [^11^C]glyburide was determined by counting an aliquot of the product solution in a dose calibrator for radioactivity and integration of the UV peak associated with the radioactive peak for comparison with a pre-determined calibration curve of glyburide. Identity of the radioactive species was confirmed by co-injection of the radioactive product with a sample solution of glyburide and co-elution of the UV and radioactive peaks.

The average radiochemical yield of [^11^C]glyburide was 5.7% based on trapped [^11^C]methyl triflate activity, with radiochemical purity of >98% and average molar activity of 22.5 Ci/µmol at the end of synthesis (n = 2).

### PET scan

PET scan was performed according to our previous reported methods with modifications 17, 18. Briefly, rats were sedated with isoflurane (3%) in a sedation chamber and kept anesthetized with isoflurane (1.5-2.5%). PET images were acquired using the Siemens FOCUS 220 PET scanner (Siemens Preclinical Solutions, Knoxville, TN) with a reconstructed image resolution of ~2 mm. Following a transmission scan, ^11^C-glyburide was injected intravenously. List-mode data were acquired and dynamic scan data were reconstructed with a filtered back projection algorithm with corrections for attenuation, normalization, scatter and randoms. The left and right brain regions of interest (ROIs) were manually drawn based on the PET image. Regional time-activity curves (TACs) were generated for the left and right brain hemispheres.

### Middle Cerebral Artery Occlusion (MCAO) model

Male Wistar rat (Charles River Laboratories), ~200 g each, and male C57BL/6 mice (Charles River Laboratories), ~20 g each, were given free access to food and water before all experiments. All animal experiments were approved by the Yale University Institutional Animal Care and Utilization Committee. MCAO models were generated according to methods that we recently reported 19-23. Briefly, for establishment of mouse MCAO models, the animals were anesthetized with 5% isoflurane (Aerrane, Baxter, Deerfield, IL) in 30% O_2_/70% N_2_O using a Tabletop Anesthesia system (Harvard Apparatus, USA). Isoflurane was then maintained at 1.5%. During the procedures, the body temperature of mice was maintained at 37.0 ± 0.5 °C. Regional cerebral blood flow (rCBF) was monitored using a laser Doppler flowmeter (AD Instruments Inc.) during the course of surgery. Mice were placed in the supine position, and a middle neck incision was made under a dissecting microscope (Leica A60). The right common carotid artery (CCA), external carotid artery (ECA), and internal carotid artery (ICA) were carefully exposed and dissected from the surrounding tissue. Then, a small hole in the ECA was made using Vanes-style spring scissors. A 6-0 silicon-coated mono-filament suture (Doccol) was introduced into the ECA and gently advanced from the lumen of the ECA into the ICA at a distance of 8-10 mm beyond the bifurcation to occlude the origin of middle cerebral artery. Successful MCA occlusion was confirmed by a reduction of rCBF by over 80%. The occlusion lasted 90 min and the monofilament was withdrawn to allow for reperfusion. For establishment of rat MCAO models, similar procedures were used except that a 4-0 silicon-coated mono-filament suture (Ducal Corporation) was used and occlusion time was 6 hours.

### TTC Staining

After euthanasia, the brains were isolated, frozen at -20 °C for 30 min, and sliced into 6 coronal slices (2 mm thick). The brain slices were then incubated with 2% triphenyltetrazolium chloride (TTC) in PBS solution at 37°C for 15 min and fixed in 4% paraformaldehyde.

### Extraction and identification of BA

*E. ulmoides* powder (50 g) was soaked in 400 mL of Dichloromethane (DCM) for two days. After filtration, the DCM extract was obtained, concentrated and emulsified with superparamagnetic iron oxide (SPIO) nanodots using the standard emulsion procedures as descried in our previous reports 24, 25. SPIO-encapsulated NPs were collected using a magnet, after which, SPIO-encapsulated NPs were re-dissolved in DCM. SPIO nanodots were removed using magnetic force. From these procedures, materials allowing for drug encapsulation were obtained. The resulting materials were separated using a silicon column (solvent: CHCl_3_:MeOH, 97:3, *v/v*), different fractions were evaluated for NP formulation. Through these procedures, we obtained one compound. ^1^H-NMR, ^13^C-NMR, and mass spectrometry analyses identified it to be BA.

### Synthesis of BA NPs

BA NPs in size of 300nm (R300) were synthesized using the standard emulsion procedures 24, 25. For typical synthesis of BA NPs encapsulated with hydrophobic cargos, including SPIO, IR780, rhodamine B (RhoB), and glyburide, the selected cargo was dissolved together with 5 mg BA in mixed organic solution of DCM (0.95 ml) and Methanol (0.05 ml), and added dropwise to a solution of 4 ml 2.5% PVA (aqueous phase). The resulting emulsion was sonicated on ice for 40 s (5s on, 5s off) and added to a stirring solution of 0.3% PVA in water (aqueous phase, 50 ml). After evaporation at 4°C overnight, BA NPs were collected by centrifugation at 18,000 rpm for 30 min. Then, the pellets were suspended with 40 ml of water, and collected by centrifugation at 18,000 rpm for 30 min to obtain the NP pellets. Finally, the pellets were suspended with 5 ml of water, sonicated for 3 min, and then lyophilized for storage. For R150, 5mg BA was dissolved in EA (0.95ml) and Methanol (0.05ml), emulsified with 4 ml of 2.5% PVA, then sonicated on ice for 40 s (5s on, 5s off) and added to a stirring solution of 0.3% PVA in water (50 ml). After evaporation at 4°C overnight, NPs were collected same as the R300. For R700, 3mg BA was dissolved in 1ml EA, then mixed with 2ml of NaOH (pH=10.0), then the mixed solution was kept static in cold room for 6 hours, then evaporated at room temperature, NPs were collected by centrifugation at 5,000 rpm for 30 min for two times after evaporation. Finally, the pellets were suspended with 5 ml of water, sonicated for 3 min, and then lyophilized for storage.

### Transmission Electron Microscopy (TEM)

NPs resuspended in 10 μL water were applied to holey carbon-coated copper grids (SPI, West Chester, PA, USA). A filter paper was used to absorb the NPs after 5 min. The grids were left at fume hood until completely dried and then visualized by using a JEOL 1230 transmission electron microscope (JEOL Ltd., Japan) at 100 kV.

### Scanning Electron Microscopy (SEM)

Samples were mounted on carbon tape and sputter-coated with gold, under vacuum, in an argon atmosphere, using a sputter current of 40 mA (Dynavac Mini Coater, Dynavac, USA). SEM imaging was carried out with a Philips XL30 SEM using a LaB electron gun with an accelerating voltage of 10 kV. The mean diameter and size distribution of the particles were determined by image analysis using image analysis software (ImageJ, National Institutes of Health). These micrographs were also used to assess particle morphology.

### Dynamic light scattering (DLS)

The hydrodynamic diameter of NPs was measured using dynamic light scattering. A transparent cuvette was filled with NPs in HPLC-grade water. The capped cuvette was placed in a Zetasizer (Malvern), and dynamic light scattering data or zeta potential was read.

### Fluorescent imaging

Mice with successful MCAO surgery were prepared. Immediately after surgery, IR780-loaded BA NPs were administered intravenously through the tail vein. Doses for each group were adjusted according to the fluorescence intensity to ensure that each mouse received the same amount of dye. Twenty-four hours later, mice were sacrificed to isolate the brain and other organs, and imaged by IVIS imaging system (Xenogen) with excitation wavelength of 745 nm and emission wavelength of 820 nm for free IR780 or IR780- loaded NPs. Fluorescence intensity in each brain was quantified using Living Image 3.0 (Xenogen).

### Identification of transporters/receptors

Plasmids for expression of IGF-1R (#11212), CD36 (#55405), GLU2 (#18086), GLU4 (#18087), and CB1 (#13391) were obtained from Addgene. ASBT cDNA was obtained from R&D Systems and cloned into pCDH-MCS-T2A-Puro for expression. TGR5 expression plasmid, pIRESneo3-mTGR5 26, was kindly provided by Dr. Wendong Huang. Plasmids were transfected into HEK293T cells by using Fugene 6 (Promega). After 24 hours, cells were treated with 100 μg/ml BA NPs which were freshly synthesized and encapsulated with 0.5 wt % Coumarin 6 (Sigma) for 1 day. Flow cytometry analysis was performed according to the standard procedures as previously described 27. Relative transcytosis ability was expressed as the ratio of the percentile of Coumarin 6 positive population in cells expressing a given molecule to the percentile in control cells.

### *In vitro* BBB model and inhibition study

The *in vitro* BBB model was established by culturing human brain microvascular endothelial hCMEC/D3 cells on the top of insert membrane and human astrocyte NHA cells at the bottom membrane in a 24-well transwell culture plate 28. After *in vitro* BBB model was successfully set up, upper chamber cells were pre-treated with CB1 inhibitor SR141716A (1μM) or vehicle solution for 1 h, then coumarin 6 (C6)-loaded BA NPs (100 μg/ml) were added into the upper chamber. 100 μl medium in lower chamber were taken out at 1h, 2h, 4h, 8h, and 24h, the total amounts of dye were quantified based on fluorescence using a BioTek microplate reader.

### *In vivo* blocking study

Mice with successful MCAO surgeries were randomly divided into 2 groups (n = 3 for each group), which received treatment of PBS and SR141716A, respectively. Thirty minutes later, IR780- loaded BA NPs were administered intravenously through the tail vein. Twenty-four hours later, mice were sacrificed to isolate the brain and imaged.

### Cignal™ reporter assay for Nrf2 activity

Luciferase-based Nrf2 activity reporter and control constructs were obtained from Qiagen and co-transfected with Renilla luciferase- expressing construct pGL4.74 (Promega) to HEK293 cells using Fugene 6 transfection reagent (Promega). After treatment with BA NPs (100 μg/ml) for 48 h, expression of firefly and Renilla luciferase were determined using a Dual-Luciferase® Reporter Assay System kit (Promega). The activity of Nrf2 signaling in cells, which was measured by the intensity of firefly luciferase, was normalized based on the intensity of Renilla luciferase.

### Western Blot

To determine the anti-oxidant effect of BA NPs on cells, NHA were randomly divided into 4 groups, which were treated with PBS, 2 μg/ml BA NPs, 10 μg/ml BA NPs and 30 μg/ml BA NPs. After 24 h, cells were lysed in RIPA lysis buffer containing protease for 30 min on ice. The protein concentration of each cell lysate sample was determined using the BCA and adjusted to equivalent amounts. Western blot analysis was performed according to the standard procedures as described in our previous report 29, using antibodies targeting Nrf2 (Novus Biologicals), HO-1 (Novus Biologicals), and beta-actin (#643802, BioLegend). To determine the anti-oxidant effect *in vivo*, mice with successful MCAO surgery were randomly divided into 2 groups (n = 3 for each group), which received treatment of PBS or 2mg BA NPs, respectively. After 24 h, the brains were harvested, and the right hemispheres containing the ischemic area were excised. The brains from normal mice without surgery were used as controls. Western blot analysis was performed as described above.

### Quantification of cellular reactive oxygen species (ROS)

NHA cells were plated in two 96-well culture microplates. After 24 hours, the cells were divided into two groups and treated with BA NPs or PBS, respectively. One plate was incubated in a hypoxia chamber with 1% O_2_. Another plate was culture in the normoxic oxygen condition (21% O_2_). Twenty-four hours later, the cells were collected and subjected to 2',7' -dichlorofluorescin diacetate (DCFDA)-based ROS assay using a DCFDA Cellular ROS Detection Assay Kit according to the manufacture's instruction (Abcam).

### *In vitro* drug release

Gly- BA NPs (3 mg) were suspended in 1 ml buffer and incubated at 37^°^C with gentle shaking. At each sampling time, NPs were centrifuged for 10 min at 12,000 rpm. The supernatant was collected and 1 ml buffer was added for continuously monitoring of the release. The amount of glyburide in supernatant was quantified by HPLC.

### Determination of the therapeutic benefits

For characterization of the treatment with BA NPs, mice with successful MCAO surgeries were randomly divided into 4 groups (n = 3), which received treatment of PBS, Free BA, 0.5 mg (25 mg/kg) BA NPs, 1.0 mg (50 mg/kg) BA NPs and 2.0 mg (100 mg/kg) BA NPs, respectively, at 0, 24 and 48 h after surgery. Three days later, the mice were sacrificed, and the brains were excised, sectioned, and stained with TTC. The infarct area in each slice was quantified using ImageJ. The infarct volume was calculated by the formula described as: Corrected infarct volume (%) = (contralateral hemisphere volume - non-infarcted ipsilateral hemisphere) / contralateral hemisphere volume × 100.

For characterization of the treatment with Gly-NPs, mice with successful MCAO surgery were randomly divided into 4 groups (n = 5 for each group), which received treatment of PBS, blank BA NPs, Gly-NPs at a dose equivalent to 5 μg/kg of glyburide, and the same amount of free glyburide, respectively. Mice were given treatment intravenously at 0, 24 and 48 h after surgery. Mice were monitored for survival for 10 days and were euthanized if one of the following criteria was met: (1) the mouse's body weight dropped below 15% of its initial weight, or (2) the mouse became lethargic or sick and unable to feed. For the study to determine the impact of treatments on infarct volume and neurological score, a cohort of mice were prepared (n = 5) and received the same treatments as described above. Three days later, the score of each mouse was assessed by a standard behavioral test 19, 21 and were scored as follows: (1) normal motor function, (2) flexion of torso and contralateral forelimb when animal was lifted by the tail, (3) hemiparalysis resulting in circling to the contralateral side when held by tail on flat surface, but normal posture at rest, (4) leaning to the contralateral side at rest, and (5) no spontaneous motor activity. Therapeutic evaluations were carried out using an unbiased approach; the reviewer who scored mouse function was unaware of which treatment group each mouse belonged to. After the evaluations, the mice were sacrificed, and the brains were excised, sectioned, and stained with TTC to determine the infract volume as described above.

### Statistical analysis

All data were collected in triplicate and reported as mean and standard deviation. Comparison between the groups were performed using a t-test. One-way ANOVA was used to analyze multiple comparisons by GraphPad Prism 7.0. *p < 0.05, **p < 0.01 and ***p < 0.001 were considered significant.

## Results

### Glyburide has a limited ability to penetrate the ischemic brain

Previous studies showed that glyburide has a limited ability to penetrate the BBB and intravenous administration of glyburide cannot achieve a therapeutic level in the brain 30, 31. We speculate that the unmet efficacy observed in the GAMES-RP trial was due to inadequate delivery of glyburide to the ischemic brain. To assess the brain penetrability of glyburide, we synthesized ^11^C-labeled glyburide (Figure 1A) 30, injected it intravenously to MCAO rats, and monitored its accumulation in the brain using PET. Regions-of-interest were defined on the PET scans, from which time-activity curves were determined. We found that, despite the presence of stroke (Figure 1B), there was essentially no difference in ^11^C-glyburide uptake between the ischemic and the contralateral hemispheres (Figure 1C, D), suggesting that glyburide is unable to efficiently penetrate the ischemic brain. Substantial evidence suggests that SUR1, the molecular target of glyburide, is highly expressed in cells in the neurovascular unit, including neurons, astrocytes, and oligodendrocytes, after stroke, which contributes significantly to cerebral edema 10, 32-34. Thus, further improvement in the treatment efficacy of glyburide requires enhanced delivery of glyburide beyond the BBB to allow its engagement with neurovascular cells.

### Identification of BA as a novel nanomaterial

**Figure 2:**
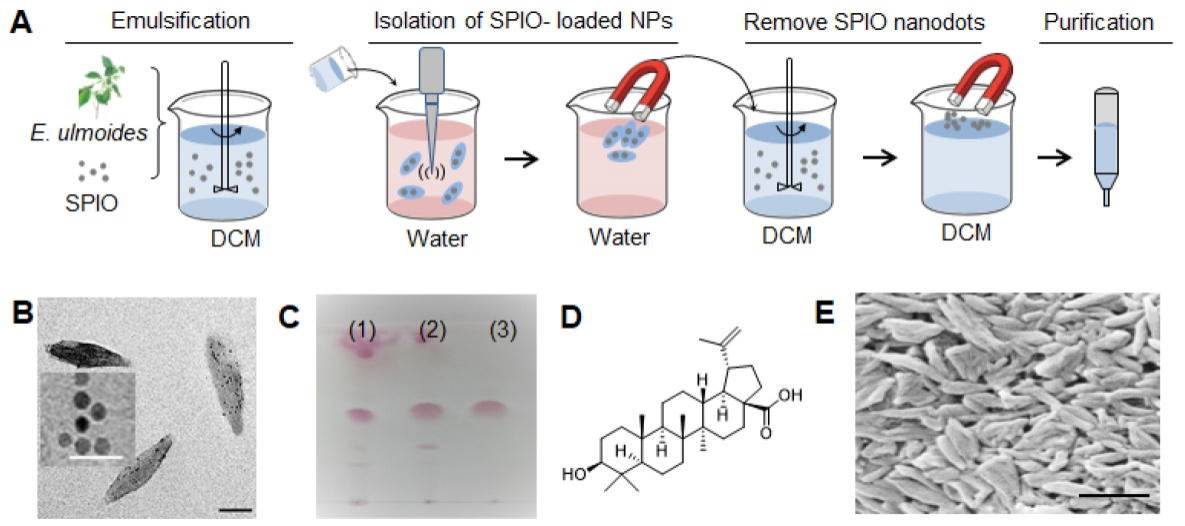
Isolation of BA from *E. ulmoides*. (A) Procedure for nanomaterial isolation. (B) Representative TEM images of SPIO (insert, scale bar 50 nm) and SPIO-encapsulated NPs. Scale bar: 150 nm. (C) TLC analysis of DCM extract (1), crude materials that enable SPIO encapsulation (2), and the selected material obtained after chromatography (3). TLC condition chloroform: methanol =95:5 (v/v); Chromogenic reagent: alcoholic solution of sulfuric acid (5%). (D) Molecular structure of BA. (E) A representative SEM image of BA NPs. Scale bar: 500 nm.

To determine whether certain medicinal herbs contain natural nanomaterials, we developed a chemical extraction approach and analyzed *E. ulmoides*. In order to isolate nanomaterials that enable drug encapsulation, we employed hydrophilic SPIO nanodots, which we previously developed 35, as the payload (Figure 2A).

First, we prepared an extract of *E. ulmoides* by soaking it in DCM. Next, we filtered and emulsified the extract with SPIO to form NPs. SPIO-encapsulated NPs were then collected using a magnet. Successful encapsulation of SPIO was confirmed by transmission electron microscope (TEM) (Figure 2B). After lyophilization, the SPIO-encapsulated NPs were dissolved in DCM. Free SPIO was removed by magnetization. The resulting extractant was separated using column chromatography. Different fractions were evaluated for NP formulation and characterized by thin layer chromatography (TLC) (Figure 2C). After these steps, we obtained our final compound, which was identified to be BA with high purity by ^1^H-NMR, ^13^C-NMR, mass spectrometry, and HPLC (Figure 2D, Figure S1). Through standard emulsion procedures, BA formed rod-shaped NPs with a length of ~315 nm and diameter of ~60 nm, or 315(l) x 60(d) nm, as determined by scanning electron microscope (SEM) (Figure 2E). DLS analysis showed that BA NPs have an average hydrodynamic diameter of 535.5 nm (Figure S2) and bear a negative charge with zeta potential of -21 mV.

### BA NPs for drug delivery to the ischemic brain

**Figure 3:**
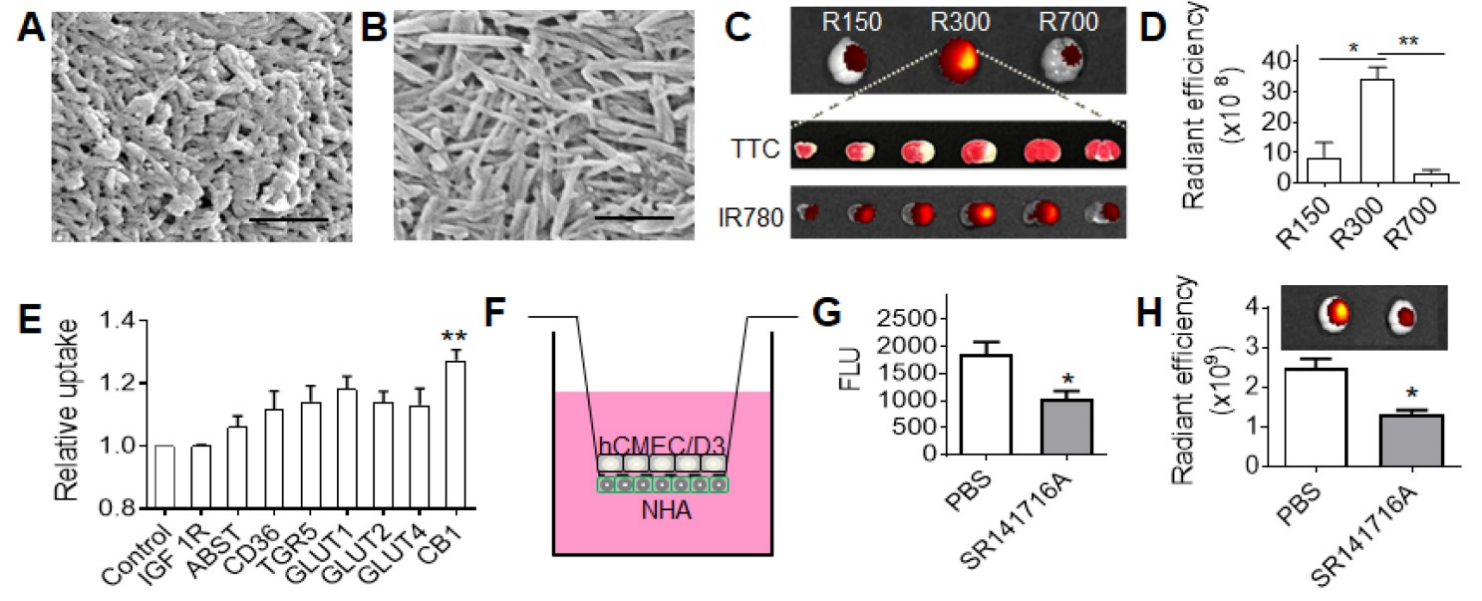
BA NPs for drug delivery to stroke. (A,B) Representative SEM images of BA NPs in 156(l) x 45(d) nm (A), and 730(l) x 35(d) (B). Scale bar: 500 nm. (C,D) Representative images (C) and semi-quantification (D) of BA NPs in the brains isolated from MCAO mice received the indicated treatment. (E) Flow cytometry analysis of the uptake of BA NPs in cells that were engineered to overexpress the indicated surface molecules. (F) Schematic diagram of *in vitro* BBB transcytosis assay. (G) *In vitro* analysis of the inhibitory effect of SR141716A on NP transcytosis. (H) Representative images (upper panel) and semi-quantification (bottom panel) of IR780- loaded BA NPs in the brains isolated from MCAO mice with and without pre-treatment of SR141716A. Intensities of IR780 fluorescence were quantified using Living Image 3.0.

BA NPs (Figure 2E) were synthesized using DCM as the solvent, water as the aqueous phase, and 4°C as the evaporation temperature. We found that the shape and size of BA NPs was tunable by varying organic phase, aqueous phase, and evaporation temperature. When a combination of ethyl acetate (EA) (solvent), water (aqueous phase), and 4°C (evaporation temperature) was used, we obtained BA NPs with a size of 156(l) x 45(d) nm (Figure 3A). When a combination of EA (solvent), NaOH solution (aqueous phase), and 25 °C (evaporation temperature) was used, we obtained BA NPs with a size of 730(l) x 35(d) (Figure 3B). To simplify the nomenclature, we designated BA NPs with a size of 156(l) x 45(d) nm, 315(l) x 60(d) nm, and 730(l) x 35(d), as R150, R300, and R700, respectively.

We evaluated R150, R300, and R700 for drug delivery to the ischemic brain. NPs were synthesized with encapsulation of IR780, a near-infrared dye, and administered intravenously to MCAO mice. The amount of R150, R300, or R700, all loaded with IR780 by ~0.9% by weight, given to each mouse was normalized to ensure each received the same amount of fluorescence. After 24 hours, the mice were euthanized, and their brains harvested and imaged. We found that, among the three tested NPs, R300 demonstrated the greatest efficiency to accumulate in the ischemic region (Figure 3C), which was 4 times and 10 times greater than that of R150 and R700, respectively (Figure 3D). In addition to its high efficiency, R300 also demonstrated a great specificity to the ischemic region. The location of ischemia, identified by TTC staining (white), had high overlap with the location of R300, identified by fluorescence of IR780 encapsulated by R300 (red to yellow) (Figure 3C). Based on these results, R300 were selected for further investigation and are referred as BA NPs for the remainder of this study. Biodistribution analysis showed that the accumulation of R300 in the brain was 1.2-fold greater than that in the liver (Figure S3A,B). We characterized the pharmacokinetics of BA NPs in mice. BA NPs loaded with rhodamine B (RhoB) were synthesized intravenously injected into mice. The blood was then collected at various time points. The concentration of RhoB in the plasma was determined and plotted versus time (Figure S3C), based on which the half-life of BA NPs in the circulatory system was calculated as 11 h. We found that BA NPs have limited toxicity to NHA cells (Figure S3D).

We explored if any transports or receptors mediate the penetration of BA NPs into the brain. Analysis by MetaDrug (Thomson Reuters) predicted that BA may interact with several surface molecules, including insulin like growth factor 1 receptor (IGF-1R), apical sodium-bile acid transporter (ASBT), CD36, TGR5, glucose transporters (GLU1, 2, 4), and cannabinoid receptor 1 (CB1). To determine if any of them interact with BA NPs, we overexpressed the candidate molecules in HEK293 cells and then added BA NPs encapsulated with C6. HEK293 cells were utilized in the initial screen in that the cells can be engineered for expression of transgenes with high efficiency 36. Twenty-four hours later, the cells were collected. The uptake of BA NPs in cells was determined by flow cytometry. We found that cells overexpressed with CB1 demonstrated the greatest efficiency (Figure 3E). This suggests that the CB1 receptor, which is primarily expressed in the central nervous system 37 and overexpressed in the ischemic brain (Figure S4) 38, may mediate the transport of BA NPs into the brain. To study the role of CB1 in NP transcytosis, we established a Transwell system as an *in vitro* model of the BBB by seeding astrocytes and endothelial cells on the basolateral and apical side, respectively (Figure 3F). When transepithelial/transendothelial electrical resistance (TEER) values reached around 100 Ω, SR141716A, a cannabinoid CB1 receptor blocker 39, was added to the upper chambers. One hour later, C6-loaded BA NPs were added. After 24 hours, the amount of NPs in the medium in the bottom chamber was determined. We found that pre-treatment with SR141716A inhibited the transcytosis of BA NPs by 44% (Figure 3G). To further confirm this finding, we established stroke mice, which received intravenous administration of SR141716A. Thirty minutes later, mice were treated with IR780-loaded BA NPs. After 24 hours, the accumulation of BA NPs in the brain was imaged and quantified based on the fluorescence of IR780. Consistent with the *in vitro* finding, we found that blocking CB1 reduced the uptake of BA NPs by 34% (Figure 3H).

Taken together, these data suggest that BA NPs penetrate the ischemic brain through CB1-mediated transcytosis in addition to BBB leakage, and the penetration efficiency is determined by their physical properties including size and shape, as well as their interaction with CB1.

### BA NPs promote stroke recovery as an antioxidant agent

**Figure 4:**
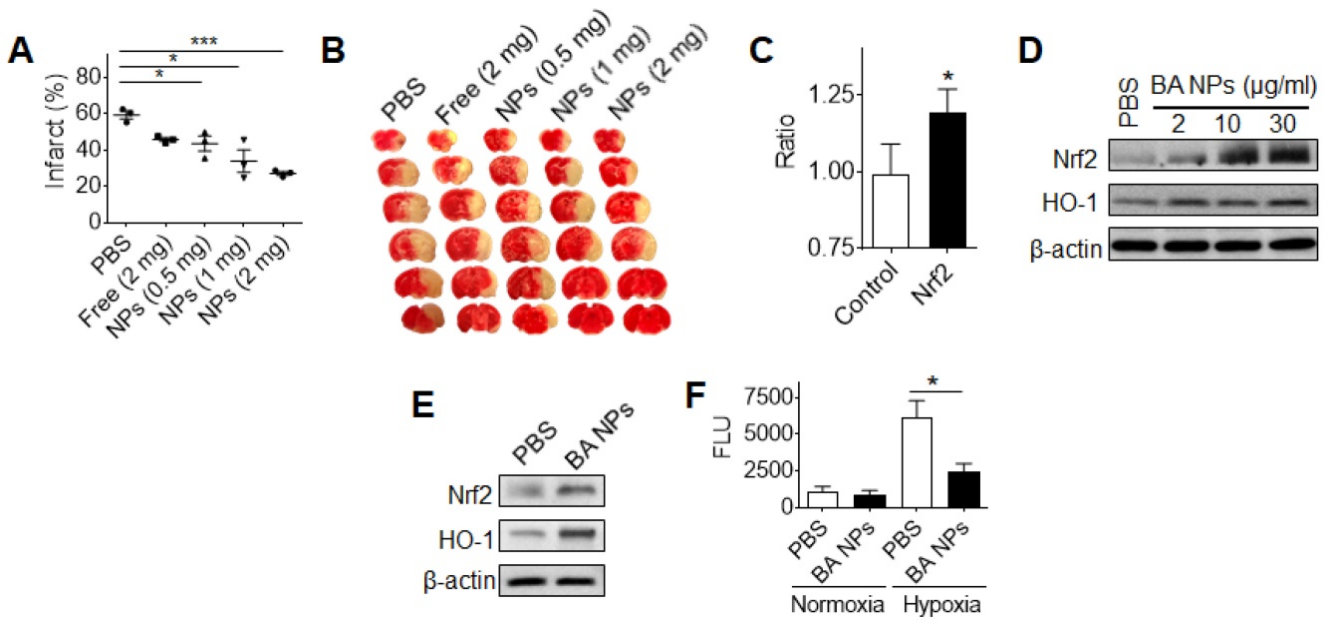
Characterization of BA NPs for stroke treatment. (A,B) Quantification (A) and representative images (B) of brain infarction in MCAO mice received treatment of BA NPs at the indicated dose. (C) The impact of BA NP treatment on the Nrf2 pathway. (D,E) Western Blot analysis of BA NP- treated astrocytes (D) and ischemic brain tissues isolated from mice (E) with and without BA NP treatment. (F) Characterization of the inhibitory effect of BA NPs on ROS production by DCFDA assay.

We evaluated BA NPs for stroke treatment. Stroke mice were established and received intravenous injection of 0.5, 1, or 2 mg BA NPs at 0, 24, and 48 hours after surgery. At day 4, the mice were euthanized. Their brains were isolated and subjected to TTC staining. We found that intravenous administration of BA NPs effectively reduced the infarct volume in a dose-dependent manner and reduced the infarct volume by 54% at the dose of 2 mg (Figure 4A,B).

Previous studies have shown that free BA has excellent antioxidant properties 40, 41. To determine if the observed anti-stroke efficacy of BA NPs can be attributed to these properties, we used the Cignal™ reporter system and examined the effect of BA NP treatment on the Nrf2 pathway, a major antioxidant response pathway 42.

We found that treatment with BA NPs significantly elevated the activity of Nrf2 signaling (Figure 4C). To confirm that BA NPs regulate the Nrf2 pathway, we analyzed human astrocytes (NHA), an astrocyte cell line that is often used in neuroscience studies 43, treated with BA NPs, as well as brain tissues isolated from mice that received intravenous administration of BA NPs. We found that, in both conditions, treatment with BA NPs significantly up-regulated the expression of both Nrf2 and heme oxygenase-1 (HO-1), a Nrf2- regulated antioxidant enzyme (Figure 4D,E). To provide further evidence that BA NPs regulate the antioxidant pathway, we exposed NHA cells with and without treatment with BA NPs to hypoxia (1% O2). Twenty-four hours later, the cellular ROS level was quantified by the standard DCFDA assay. We found that treatment with BA NPs significantly inhibited ROS production in response to hypoxia exposure (Figure 4F).

Collectively, these results suggest that systemic treatment with BA NPs promotes stroke recovery through regulation of the antioxidant pathway.

### BA NPs enhance the delivery and efficacy of glyburide for stroke treatment

**Figure 5:**
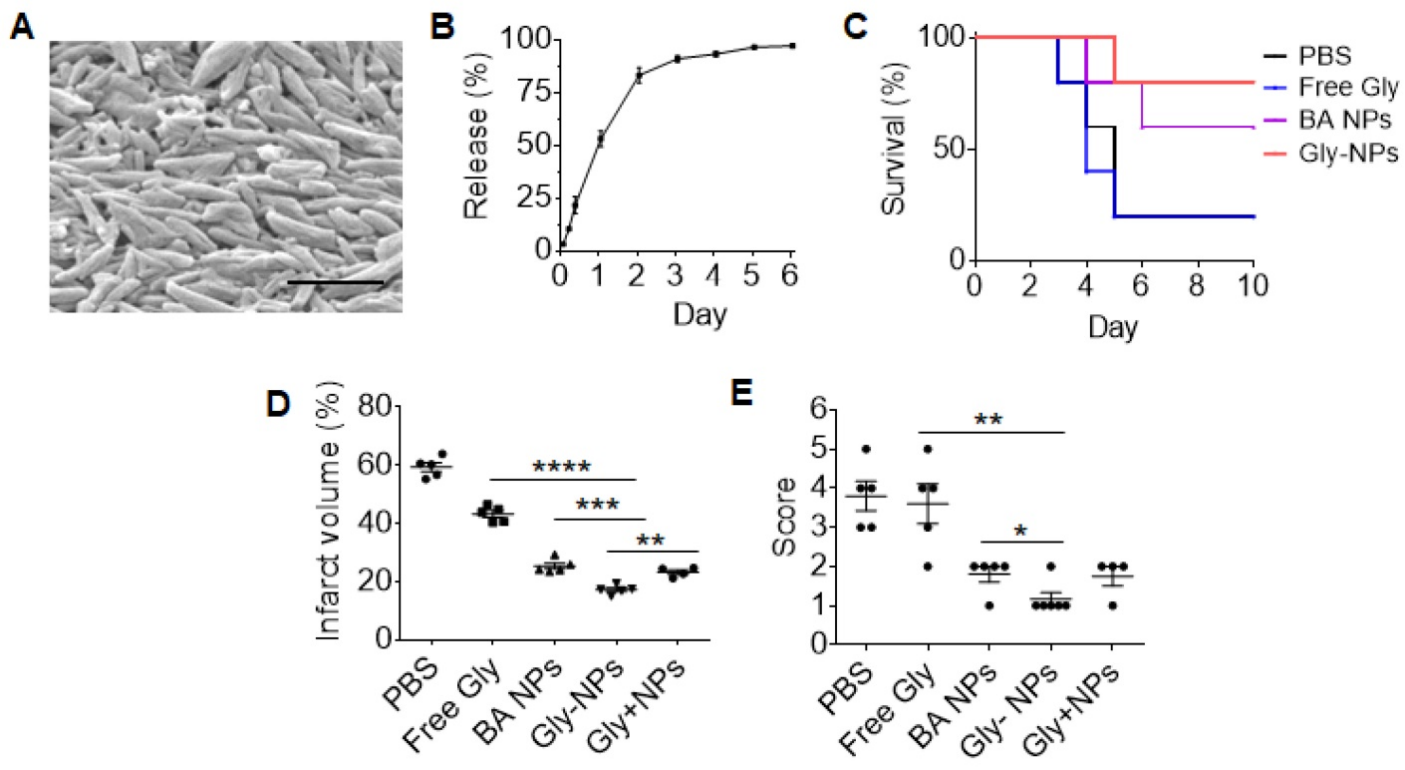
Characterization of the pharmacological activities of Gly-NPs for stroke treatment. (A) A representative SEM images of Gly-NPs. Scale bar: 500 nm. (B) Release of glyburide from Gly-NPs in PBS at 37 °C. (C-E) Kaplan-Meier survival analysis (C), infarct volume (D, day 3 after surgery), and neurological scores (E, day 3 after surgery) of MCAO mice receiving the indicated treatments (n = 5).

We explored BA NPs as a drug carrier for intravenous delivery of glyburide for stroke treatment. BA NPs were synthesized with encapsulation of glyburide. Although it is a potent drug for stroke treatment 10, glyburide may induce hypoglycemia at a high dose. To deliver 2 mg BA NPs, we limited the loading of glyburide to 0.005% by weight (0.05 ug glyburide per 1 mg NPs). The resulting NPs, referred to as Gly-NPs, were characterized for their physical properties and drug release. Analysis by SEM showed that encapsulation of glyburide did not alter the morphology of BA NPs (Figure 5A). Control release studies found that 91% of glyburide was released from Gly-NPs over three days (Figure 5B). Next, we evaluated Gly-NPs for stroke treatment. MCAO mice were established and received intravenous administration of Gly-NPs at a dose equivalent to 5 µg/kg of glyburide per injection 0, 24, and 48 h after surgery. We found that treatment with Gly-NPs at glyburide equivalent dose of 5 µg/kg did not induce hypoglycemia (Figure S5A) but significantly improved mouse survival (p < 0.01, Figure 5C), reduced infarct volumes by 36% (Figure 5D, Figure S5B) and improved neurological scores (Figure 5E). In contrast, treatments with the same amount of BA NPs or glyburide alone showed significantly less efficacy. We found that the therapeutic benefits of Gly-NP treatment could not be achieved simply through treatment with a mixture of the same amount of glyburide and BA NPs (Gly + NPs) (Figure 5D, E), suggesting that formulation in NPs is indispensable. As expected, we found that treatment with Gly-NPs significantly reduced brain edema (Figure S5C) and reduced BBB leakage (Figure S5D).

## Discussion

Glyburide is known to have a limited ability to penetrate the BBB 30, 31. Using PET, we found that glyburide is no more efficient in penetrating the brain on the ischemic side versus the ipsilateral side (Figure 1). This finding may explain the observation in our recently completed GAMES-RP trial that although intravenous administration of glyburide enhanced patient survival, it could not significantly improve clinical outcome 11.

To enhance the delivery of glyburide to the brain, we developed a chemical extraction approach and isolated BA, a natural nanomaterial, from *E. ulmoides*. We showed that BA formed NPs, which were capable of penetrating the ischemic brain through interaction with CB1, improving functional recovery through antioxidant effects and enhancing the delivery efficacy of glyburide to the brain.

This study is significant on three major fronts. First, this study identifies a potential reason accounting for the unmet efficacy of glyburide for stroke treatment. This finding may guide the development of novel approaches to further improving the clinical efficacy of glyburide.

Second, this study reveals a new direction to discover functional nanomaterials from medicinal herbs for drug delivery. It is documented in the literature that some natural compounds, such as ursolic acid 44 and oleanolic acid 45, can form NPs. However, isolation of such nanomaterials using a chemical approach has not been reported. We demonstrated that such compounds can be isolated from a SPIO-based magnetization approach. We found that, different from most existing nanomaterials, such as polymers or lipids, which cannot penetrate the brain without additional modifications and do not have biological activity without drug encapsulation, the nanomaterials isolated from selected medicinal herbs may penetrate the brain and/or exhibit bioactivity on their own. This finding may significantly impact drug delivery research through diversification of functional nanomaterials for drug delivery and disease treatment. The simplicity of these single-component NPs is beneficial for their clinical translation.

Third, this study establishes a new formulation of glyburide, glyburide-loaded BA-NPs. BA NPs were chosen as the delivery carrier for three major reasons. First, BA NPs not only enhances the delivery of glyburide to the brain by fully capitalizing on its anti-edema properties, but also reduces its side effects. Currently in clinics, the efficacy of glyburide has been limited by a low dose (3 mg/d), as glyburide given at higher doses may induce hypoglycemia. The use of Gly-NPs reduces the exposure of glyburide to the circulatory system and thus limits the risk of hypoglycemia. Second, glyburide-loaded BA-NPs represent the simplest solution to treat both cerebral edema and oxidation, two major complementary targets that are promising stroke treatment 46. Third, administration of glyburide-loaded BA-NPs improves patient convenience when delivering glyburide. Due to its limited brain retention and short plasma half-life, current usage of glyburide requires continuous infusion for 72 hours. In preclinical animal studies, glyburide required continuous administration using osmatic pumps 10. Unlike free drugs, NPs have sizes that are optimal for longer retention in brain tissue and can provide controlled release of cargo agents over time. We found that daily injection of Gly-NPs is sufficient to generate significant therapeutic benefit.

## Conclusion

In summary, we demonstrated that glyburide has a limited ability to penetrate the ischemic brain. We developed a novel approach to discover functional nanomaterials from medicinal herbs. We established a new formulation of glyburide through encapsulation into BA NPs, which provides anti-edema and antioxidant combination therapy via a simple formulation. Due to its simplicity, multifunctionality, and significant efficacy, the resulting formulation may be translated into clinical applications to improve clinical management of stroke.

## Abbreviations

BBB: blood-brain barrier
BA: betulinic acid
NP: nanoparticle
tPA: tissue-type plasminogen activator
SUR1-TRPM4: sulfonylurea receptor 1-transient receptor potential melastatin 4
TCM: traditional Chinese medicine
MCAO: middle cerebral artery occlusion
PET/CT: positron emission tomography-computed tomography
ROI: regions of interest
TACs: regional time-activity curves
rCBF: regional cerebral blood flow
CCA: common carotid artery
ECA: external carotid artery
ICA: internal carotid artery
TEM: transmission electron microscopy
SEM: scanning electron microscopy
TLC: thin layer chromatography
TTC: triphenyltetrazolium chloride
HO-1: heme oxygenase-1
TEER: transepithelial/transendothelial electrical resistance
