# Supplementary material for: Anti-edema and antioxidant combination therapy for ischemic stroke via glyburide-loaded betulinic acid nanoparticles
Source: Theranostics. 2019 Sep 21;9(23):6991–7002. doi: 10.7150/thno.35791 (PMC6815966; doi:10.7150/thno.35791)
Supplement: Supplementary file 1 — Supplementary figures and tables. [file thnov09p6991s1.pdf]

Supplemental Information

Anti-edema and antioxidant combination therapy for ischemic stroke via glyburide- loaded betulinic acid nanoparticles

Gang Deng,[1,2] Chao Ma,[1,3] Haitian Zhao,[1,4] Shenqi Zhang,[1,2] Jun Liu,[1] Fuyao Liu,[1] Zeming Chen,[1] Ann T. Chen,[5] Xin Yang,[1,4] Jonathan Avery,[1] Pan Zou,[1] Fengyi Du,[1] Keun-poong Lim,[6] Daniel Holden,[6] Songye Li,[6] Richard E. Carson,[5,6] Yiyun Huang,[5] Qianxue Chen,[2] W. Taylor Kimberly,[7] J. Marc Simard,[8] Kevin N. Sheth,[9]* Jiangbing Zhou[1,5]*

[1]Department of Neurosurgery, [5]Department of Biomedical Engineering, [6]PET Center, Department of Radiology and Biomedical Imaging, [9]Department of Neurology, Yale University, New Haven, CT, 06510, USA. [2]Department of Neurosurgery, Renmin Hospital of Wuhan University, Wuhan 430060, China. [3]College of Biological Sciences and Biotechnology, Beijing Forestry University, Beijing 100083, China. [4]School of Chemistry and Chemical Engineering, Harbin Institute of Technology, Harbin 150090, China. [7]Department of Neurology, Division of Neurocritical Care, Massachusetts General Hospital, Boston, MA, 02114, USA. [8]Department of Neurosurgery, University of Maryland School of Medicine, Baltimore, MD, 21201, USA.

Extraction and identification of betulinic acid (BA)

BA was obtained as colorless needle crystals, ESIMS *m/z*: 455.3[M-H]$^-$ ($C_{30}H_{48}O_3$); ^{1}H-NMR (DMSO-*d*$_6$,400 MHz) δ: 4.61 (1H, s, H-29a), 4.48 (1H, s, H-29b), 2.91 (1H, dd, H-3), 2.23 (1H, dt, H-18); ^{13}C-NMR (DMSO-*d*$_6$, 100 MHz) δ: 177.9 (C-28), 151.0 (C-20), 109.9 (C-29), 77.2 (C-3), 55.9 (C-17), 55.4 (C-5), 50.4 (C-9), 49.1 (C-19), 47.1 (C-18), 42.5 (C-14), 40.7 (C-8), 38.9 (C-4), 38.7 (C-1), 37.9 (C-13), 37.2 (C-10), 36.9 (C-22), 34.4 (C-7), 32.4 (C-16), 30.7 (C-15), 29.7 (C-21), 28.6 (C-23), 27.6 (C-2), 25.6 (C-12), 20.9 (C-11), 19.4 (C-30), 18.4 (C-6), 16.4 (C-26), 16.3 (C-25), 16.2 (C-24), 14.8 (C-27). The NMR data is consistent with those in literature[1].

A

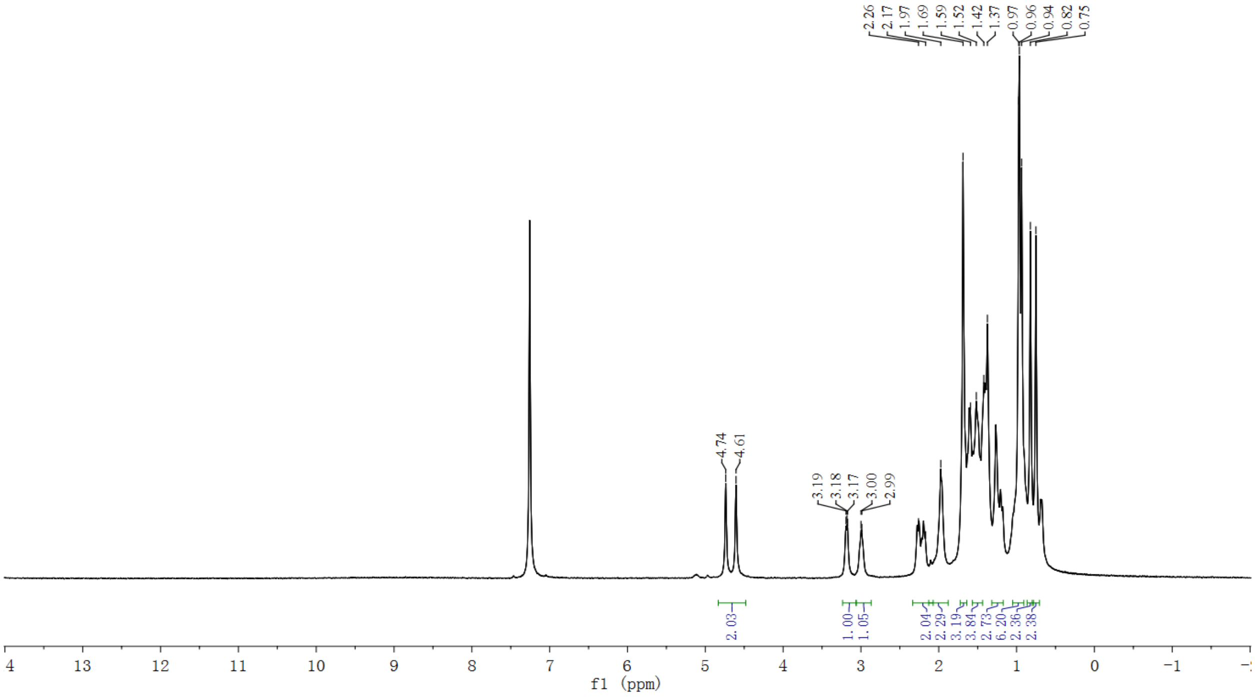

B

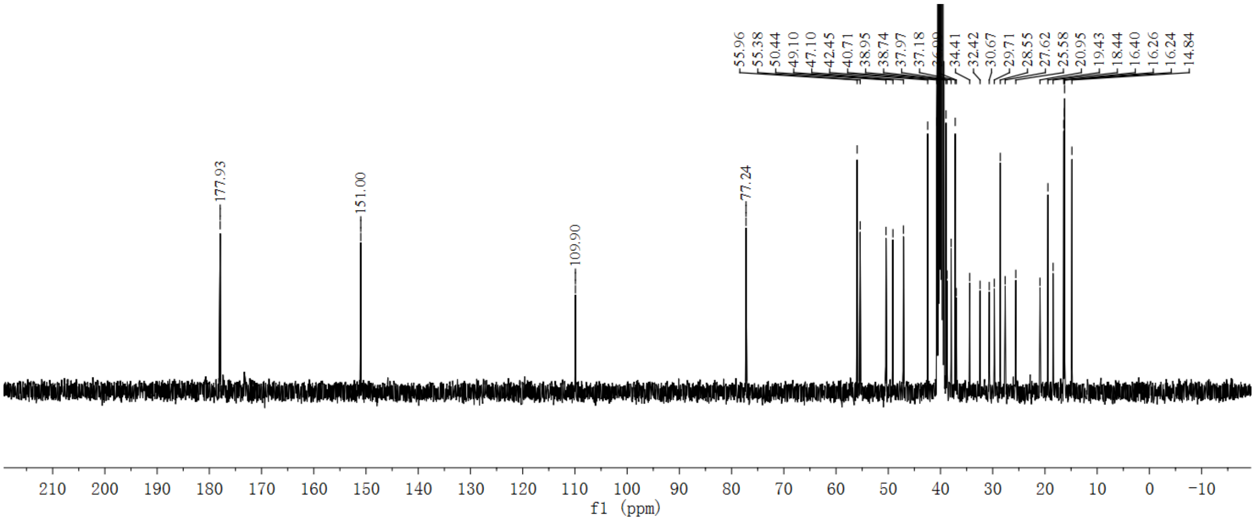

C

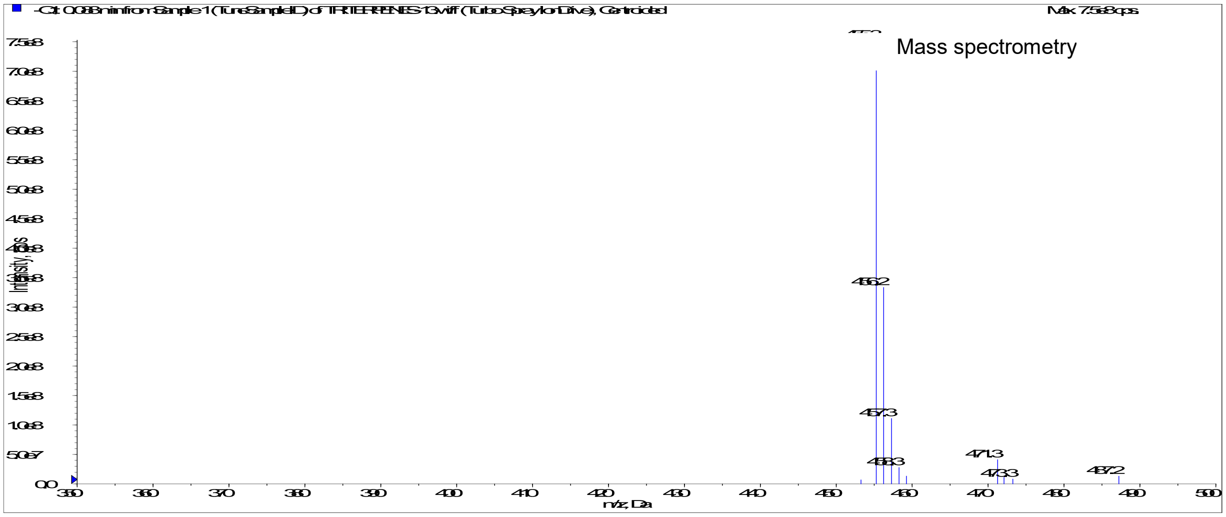

D

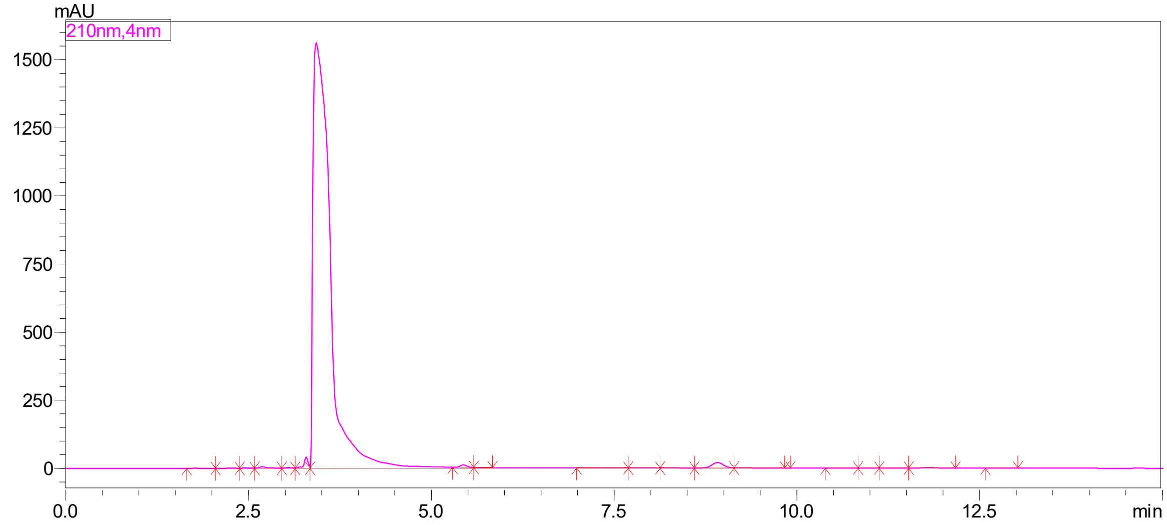

Supplementary Figure 1. Identification and characterization of betulinic acid by ^{1}H NMR (**A**), ^{13}C NMR (**B**), MS (**C**) and HPLC (**D**).

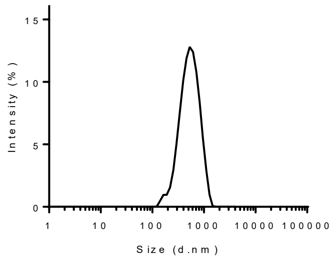

Supplementary Figure 2. Dynamic light scattering (DLS) analysis of the hydrodynamic diameter of BA NPs. For DLS analysis, 0.1 mg NPs in 1 ml ddH2O water was added to a transparent cuvette and subjected to measurement using a Zetasizer (Malvern).

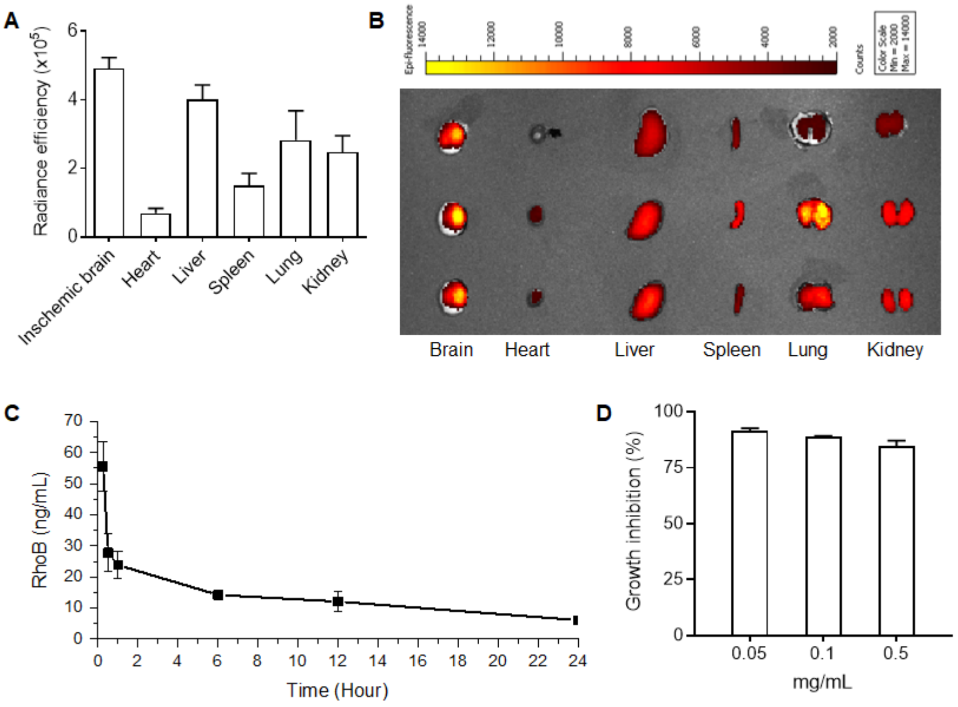

Supplementary Figure 3. Quantification (**A**) and representative images (**B**) of IR780- loaded BA NPs in major organs after intravenous administration to MCAO mice. Mice were euthanized 24 hours after treatment. Images were captured by an IVIS system. Intensities of IR780 fluorescence were quantified using Living Image 3.0. (**C**) Plasma concentrations of RhoB verse time in mice after intravenous administration of RhoB-loaded BA NPs. Mice (n = 3) received intravenous administration of RhoB-loaded BA NPs. Blood samples were collected at 15 min, 30 min, 1, 6, 12 and 24 h after injection. The plasma RhoB concentration was quantified based on RhoB fluorescence and plotted with time. (**D**) Cytotoxicity of BA NPs on NHA cells. Cells were treated with BA NPs at the indicated concentrations. Three days later, the cells were subjected to the standard MTT assay to determine the growth of cells with and without treatment.

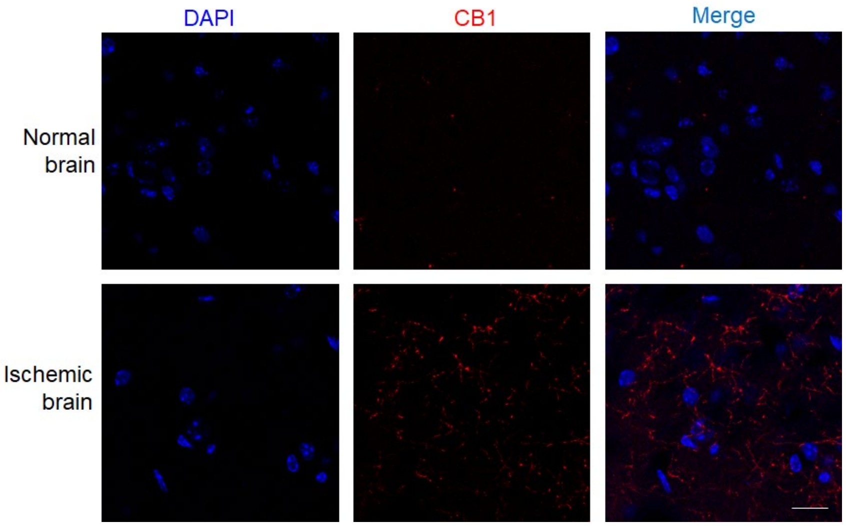

Supplementary Figure 4. Confocal analysis of the expression of CB1 in the ischemic brain. Seventy-two hours after MCAO surgery, mice were anesthetized, perfused with PBS and 4% paraformaldehyde. The brains were dissected and placed in 4% paraformaldehyde for 2 days and transferred into 30% sucrose. After fixation, the brains were sliced at 30 um by cryostat section. After blocking with 4% bovine serum albumin for 30 min and washed three times with PBS, the sections were incubated with anti-Cannabinoid Receptor 1 antibody (1:500) (ab23703, abcam) overnight at 4°C. After washing with PBS for three times, the sections were then incubated with donkey anti-Rabbit IgG (ab150075, abcam) for 30 min. The slides were mounted with DAPI mounting medium and imaged using a confocal microscope (Leica TCS SP8). Scale bar: 30 μm.

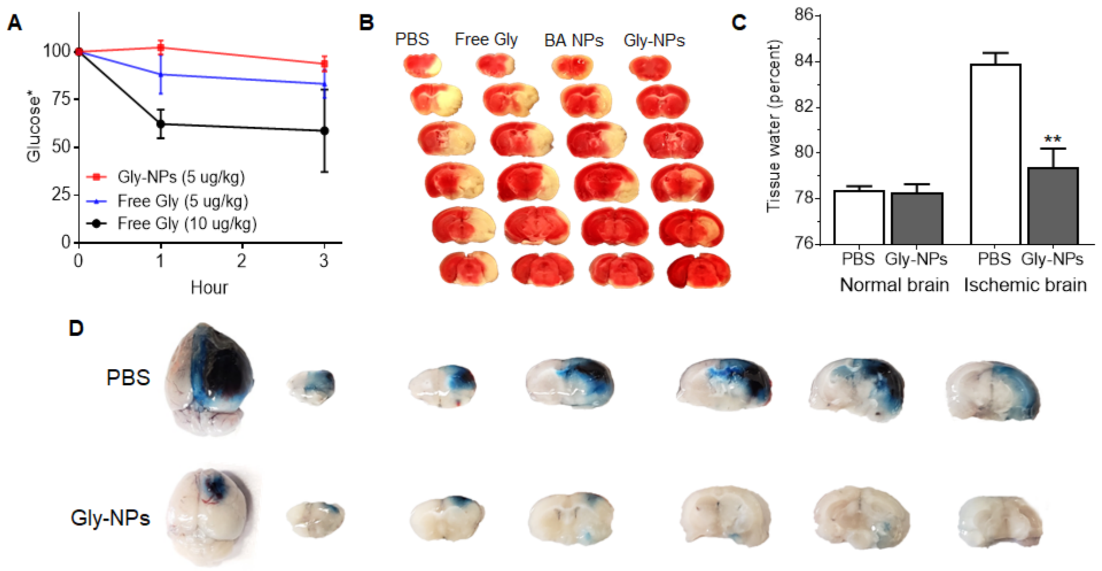

Supplementary Figure 5. Characterization of the pharmacological activities of Gly-NPs on stroke. **(A)** The effect of treatment with free glyburide or Gly-NPs on blood glucose level at the indicated doses. **(B)** Representative images of infarction in the brains isolated from MCAO mice received the indicated treatments. **(C)** Treatment with Gly-NPs effectively reduced brain edema. MCAO mice were prepared and received a single injection of PBS, or Gly-NPs at a dose equivalent to 5 μg/kg of glyburide immediately after surgery ($n = 5$). After 24 hours, the mice were sacrificed and the brains were excised, and weighted to obtain the wet weight. Then, the brains were lyophilized for 24 h and weighted to obtain the dry weight. Tissue water content was calculated as: Tissue water (%) = (wet weight-dry weight)/wet weight × 100. **(D)** Evans-blue leakage in the brain after the indicated treatment. Mice with successful MCAO surgeries were randomly divided into 2 groups ($n = 3$), which received intravenous administration of PBS and Gly-NPs at 0, 24, and 48 h after surgery. Twenty-four hours after the last injection, 2% Evans blue dye (Sigma-Aldrich, StLouis, MO, USA) in 0.9% saline (5 mL/kg) was injected into the right femoral vein of mice. After additional 12 hours, the mice were perfused. The brains and brain slices were isolated and prepared and imaged.

Reference

1. Sholichin M, Yamasaki K, Kasai R, Tanaka O. 13-C Nuclear Magnetic Resonance of Lupane-Type Triterpenes, Lupeol, Betulin and Betulinic Acid. CHEMICAL & PHARMACEUTICAL BULLETIN. 1980; 28: 1006-8.
